# Methadone Initiation in the Emergency Department for Opioid Use Disorder

**DOI:** 10.5811/westjem.18530

**Published:** 2024-08-01

**Authors:** Daniel Wolfson, Roz King, Miles Lamberson, Jackson Lyttleton, Colin T. Waters, Samantha H. Schneider, Blake A. Porter, Kyle M. DeWitt, Peter Jackson, Martha W. Stevens, John Brooklyn, Richard Rawson, Elly Riser

**Affiliations:** * University of Vermont Larner College of Medicine, Department of Emergency Medicine, Burlington, Vermont; † Rush University Medical Center, College of Medicine, Chicago, Illinois; ‡ The University of Vermont Medical Center, Department of Pharmacy, Burlington, Vermont; § University of Vermont Larner College of Medicine, Department of Psychiatry, Burlington, Vermont; ∥ University of Vermont, Vermont Center for Behavior and Health, Center on Rural Addiction, Burlington, Vermont; ¶ University of Vermont Larner College of Medicine, Department of Family Medicine, Burlington, Vermont; # University of California Los Angeles School of Medicine, Los Angeles, California; ** University of Vermont Larner College of Medicine, Department of Medicine, Burlington, Vermont

## Abstract

**Introduction:**

Overdose deaths from high-potency synthetic opioids, including fentanyl and its analogs, continue to rise along with emergency department (ED) visits for complications of opioid use disorder (OUD). Fentanyl accumulates in adipose tissue; although rare, this increases the risk of precipitated withdrawal in patients upon buprenorphine initiation. Many EDs have implemented medication for opioid use disorder (MOUD) programs using buprenorphine. However, few offer methadone, a proven therapy without the risk of precipitated withdrawal associated with buprenorphine initiation. We describe the addition of an ED-initiated methadone treatment pathway and compared its 72-hour follow-up outpatient treatment engagement rates to our existing ED-initiated buprenorphine MOUD program.

**Methods:**

We expanded our ED MOUD program with a methadone treatment pathway. From February 20–September 19, 2023, we screened 20,504 ED arrivals; 5.1% had signs of OUD. We enrolled 61 patients: 28 in the methadone; and 33 in the buprenorphine pathways. For patients who screened positive for opioid use, shared decision-making was employed to determine whether buprenorphine or methadone therapy was more appropriate. Patients in the methadone pathway received their first dose of up to 30 milligrams (mg) of methadone in the ED. Two additional methadone doses of up to 40 mg were dispensed at the time of the ED visit and held in the department, allowing patients to return each day for observed dosing until intake at an opioid treatment program (OTP). We compared 72-hour rates of outpatient follow-up treatment engagement at the OTP (for those on methadone) or at the addiction treatment center (ATC) (for those on buprenorphine) for the two treatment pathways.

**Results:**

Of the 28 patients enrolled in the methadone pathway, 12 (43%) successfully engaged in follow-up treatment at the OTP. Of the 33 patients enrolled in the buprenorphine pathway, 15 (45%) successfully engaged in follow-up treatment at the ATC (relative risk 1.06; 95% confidence interval 0.60–1.87).

**Conclusion:**

Methadone initiation in the ED to treat patients with OUD resulted in similar 72-hour follow-up outpatient treatment engagement rates compared to ED-buprenorphine initiation, providing another viable option for MOUD.

## BACKGROUND

The opioid crisis in the United States continues unabated with 106,699 drug-involved fatalities in 2021, primarily involving illicitly manufactured high-potency synthetic opioids, and is further complicated by adulterants such as xylazine and gabapentin.^1^ ^–^ ^6^ Individuals with opioid use disorder (OUD) face the highest risk of death within the first 48 hours following an ED (emergency department) visit for a non-fatal overdose.^7^ The ED identification of patients with OUD and initiation of buprenorphine treatment has proven effective and is supported by the American College of Emergency Physicians and the California Bridge network of hospitals.^8^ ^–^ ^12^ However, the rising prevalence of fentanyl in the illicit drug supply complicates treatment due to its accumulation in adipose tissue, potentially causing precipitated withdrawal upon buprenorphine initiation.^13^ ^–^ ^15^ Additionally, abstaining from fentanyl for the required pre-induction period may be difficult for some, leading them to avoid further buprenorphine or favor methadone for their medication for OUD (MOUD).^16^ ^–^ ^20^ Methadone, a synthetic full mu-opioid receptor agonist, avoids these complications, as it does not precipitate withdrawal.^21^ ^,^ ^22^ In response, the University of Vermont Medical Center (UVM) enhanced its existing Start Treatment and Recovery (STAR) program, an ED-based initiative to initiate MOUD in patients with OUD. Originally focused on buprenorphine, the program was expanded to include methadone, adapting to the shifting landscape of opioid use and patient needs. We describe the implementation of an ED-initiated methadone treatment pathway, comparing its 72-hour follow-up outpatient treatment engagement rates to our existing ED-initiated buprenorphine MOUD program.

## METHODS

**Figure 1.**
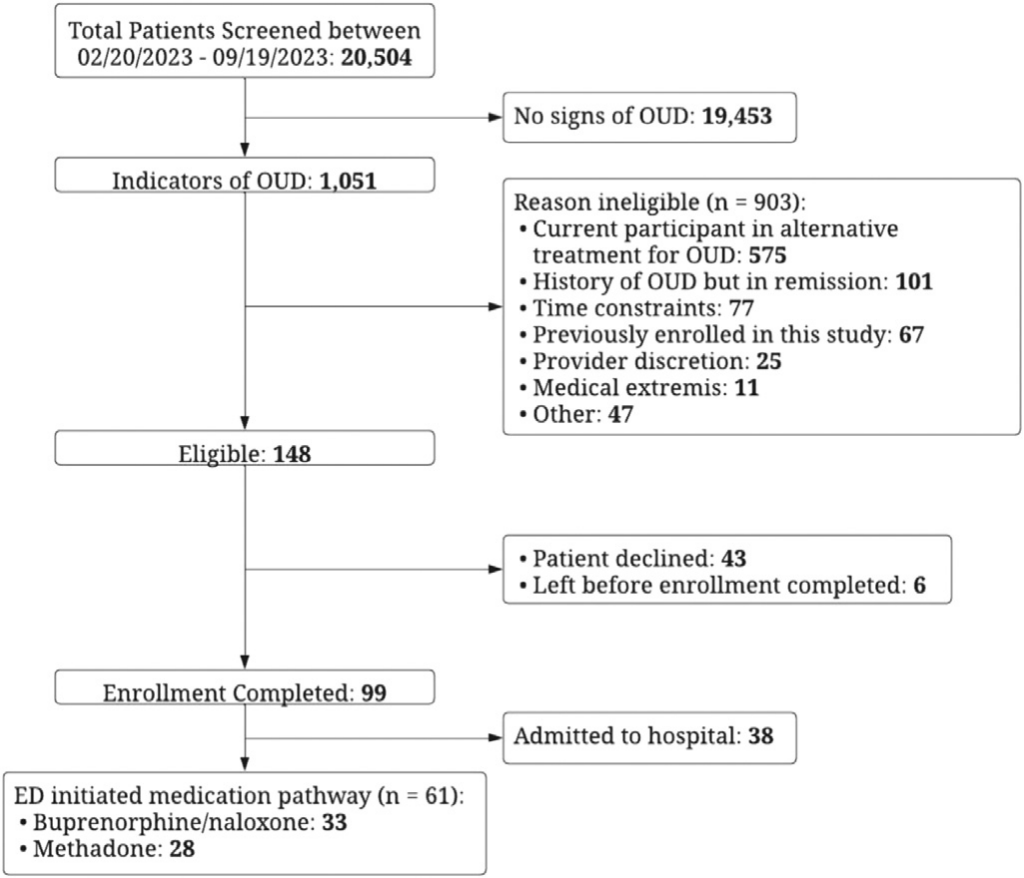
Flow chart of screening of emergency department patients and enrollment in a STAR (start treatment and recovery) program. February 20–September 19, 2023. *ED*, emergency department; *OUD*, opioid use disorder.

We performed an open trial comparing two MOUD treatment pathways where patients in the ED who met OUD criteria and agreed to treatment chose between initiation onto buprenorphine or methadone. From February 20–September 19, 2023 we screened charts of 20,504 ED arrivals, with 1,051 (5.1%) having signs of OUD. Of these, 903 were determined ineligible, 43 patients declined treatment, and six patients eloped. Patients declining participation due to time constraints or deemed unsuitable for the study at clinician discretion were excluded. Enrollment to initiate MOUD in the STAR program was completed for 61 patients with 28 initiated on methadone and 33 on buprenorphine. Not included in this analysis were an additional 38 patients who were enrolled in STAR but admitted to the hospital (Figure 1). The STAR program coordinators screened patient charts from 9 am to 9 pm daily and remained on call for enrollments 24/7. They approached identified patients to confirm opioid use, eligibility, and interest in starting MOUD.

An emergency clinician then used the *Diagnostic and Statistical Manual of Mental Disorder*s, 5^th^ Ed, criteria to confirm the diagnosis of OUD and assess readiness for treatment. Shared decision-making was used to decide on the most appropriate MOUD treatment pathway: methadone or buprenorphine/naloxone. This process involved a discussion between the clinician and the patient that included a review of the risks and benefits of each medication, the severity of OUD, the patient’s prior experience/preference, and clinical factors such as drug interactions or QT prolongation. We compared rates of 72-hour follow-up treatment engagement at the opioid treatment program (OTP) (for those on methadone) or at the UVM Addiction Treatment Center (ATC) (for those on buprenorphine) for the two treatment pathways using a chi-square test Stata/SE 18.0 (StataCorp, College Station, TX).

Patients signed a disclosure agreement allowing access to their electronic health record, which was sent to the OTP/ATC via secure email. Patients received details about their outpatient treatment appointment and were provided with transportation vouchers and a cell phone, when necessary. Patients were linked to an ED peer recovery coach who engaged with them during their ED visit and continued support through phone calls for up to 10 days following discharge.

**Figure 2.**
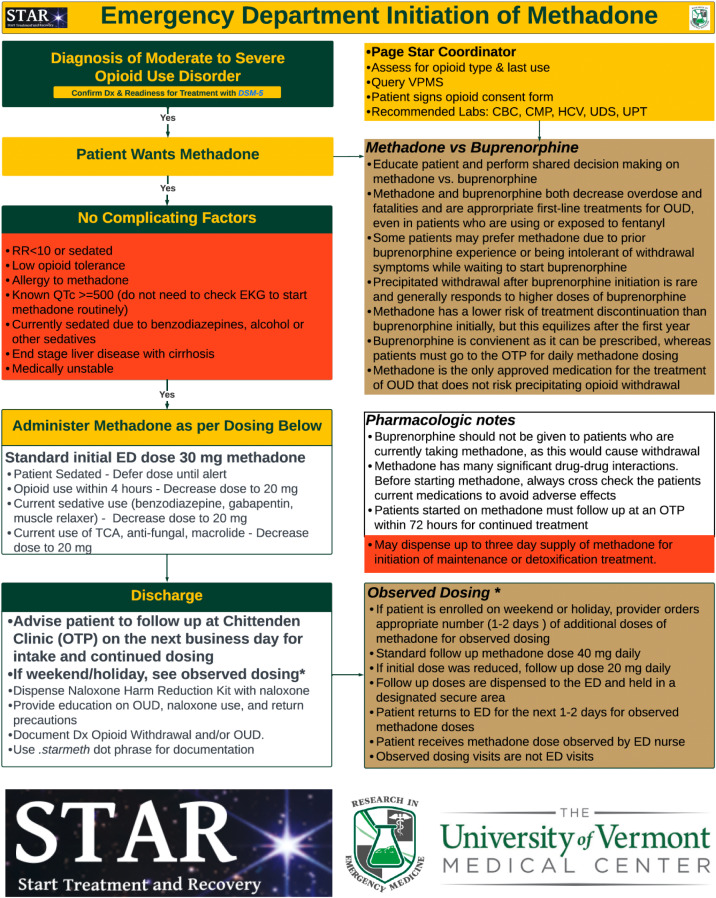
Emergency department initiation of methadone and STAR (start treatment and recovery) pathway.

**Methadone Treatment Pathway:** Patients receiving methadone were given the standard US Food and Drug Administration-recommended starting dose of 30 milligrams (mg) orally with subsequent dosing of 40 mg on the following days if bridging doses were required until the OTP appointment.^23^ The initial dose was reduced to 20 mg for patients with known opioid use in the prior four hours, if they were currently using other sedatives, or with relevant drug interactions. Methadone was not offered if the patient had a respiratory rate <10 breaths/minute, an allergy to methadone, end-stage liver disease, medical extremis, or a known QTc ≥ 500 milliseconds. Electrocardiograms were not routinely required but were obtained for risk factors in patient history or medications. Basic labs and urine drug screen were obtained. Patients were instructed to follow up at the OTP the next business day. If a patient was initiated on methadone on a weekend or holiday, the appropriate number of additional methadone doses were dispensed and held in a lock box in the ED for observed dosing. The patient was instructed to return to the ED to receive follow-up doses. Patients returning for re-dosing were not required to check in as an ED patient but were given their methadone dose, observed, and documented by a nurse using a scripted template (Figure 2).^24^

**Figure 3.**
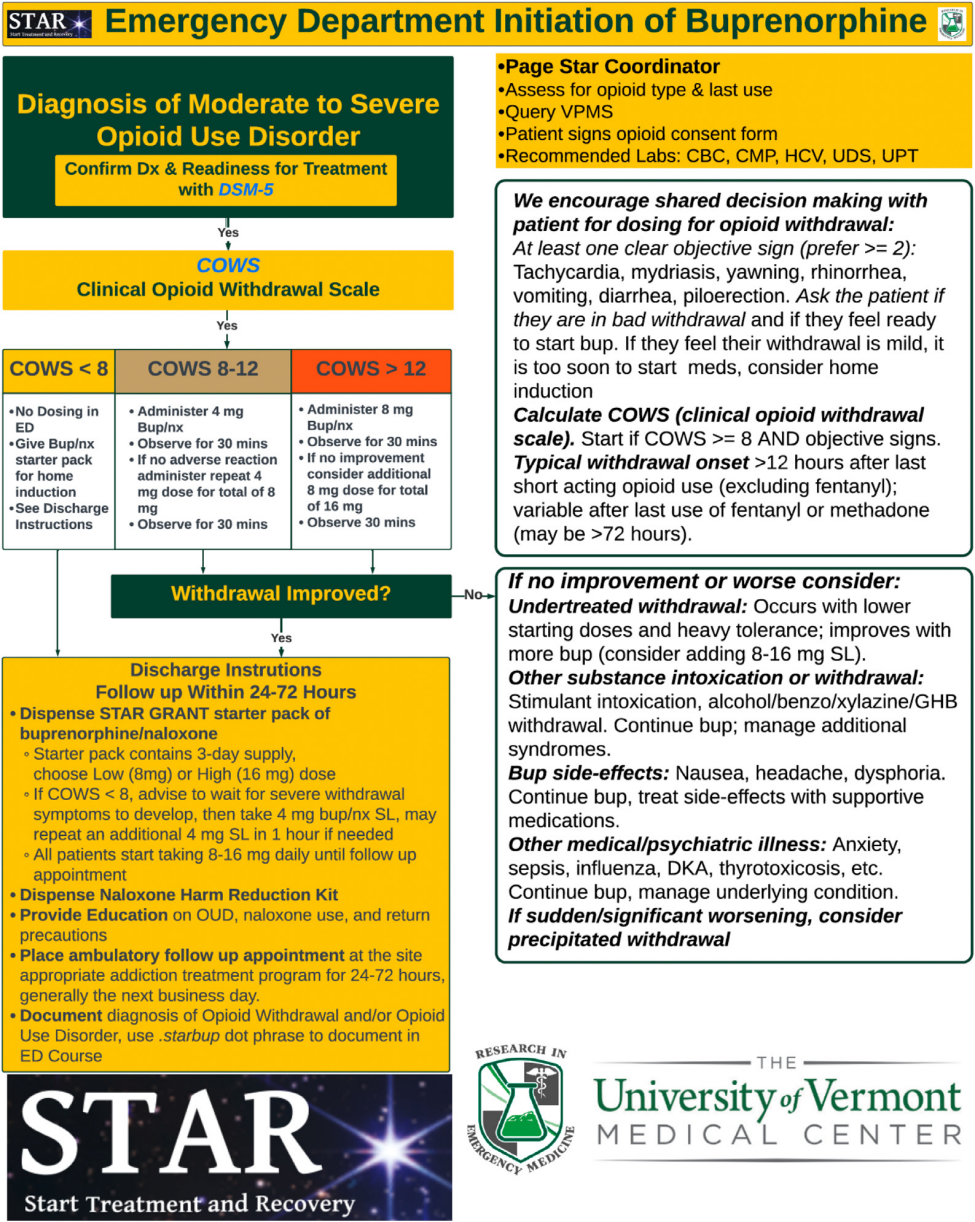
Emergency department initiation of buprenorphine STAR (start treatment and recovery) pathway.

**Buprenorphine/naloxone Treatment Pathway:** For patients receiving buprenorphine, the Clinical Opioid Withdrawal Scale (COWS) guided dosing strategies. Patients with a COWS score <8 underwent home initiation. For scores of 8–11, initiation in the ED with 8 mg buprenorphine/naloxone was provided, and for scores >12, a 16 mg dose was administered. All patients received a take-home starter pack with a three-day supply of buprenorphine/naloxone and a follow-up appointment at the ATC within 72 hours (Figure 3).^25^

**Institutional Review Board Review:** The University of Vermont Research Protections Office deemed this project to meet criteria for research not requiring review.

## RESULTS

Patients enrolled in the methadone or buprenorphine pathways had similar demographics with no significant differences between groups by gender, race, ethnicity, age, mode of ED arrival, or ED disposition. Of the 28 patients in the methadone pathway, 12 (43%) attended the OTP for ongoing methadone treatment. Of the 33 patients enrolled in the buprenorphine pathway, 15 (45%) attended the ATC for ongoing buprenorphine/ naloxone treatment. The 72-hour rates of successful follow-up outpatient treatment engagement for patients enrolled in the methadone vs buprenorphine pathways were not significantly different (relative risk 1.06; 95% confidence interval CI, 0.60–1.87).

## DISCUSSION

We enhanced our ED’s existing buprenorphine-based MOUD program by incorporating a treatment pathway for initiating methadone. Our findings show that ED-initiation of methadone for OUD is practical and achieves 72-hour follow-up outpatient treatment engagement rates comparable to those of buprenorphine. While previous case studies have documented successful initiation of methadone in the ED, we are one of the first to systematically report on the implementation of a clinical practice pathway for methadone initiation in the ED followed by linkage to ongoing care.^22^ ^,^ ^26^ Traditionally, methadone has not been used in the ED due to the potential to cause fatal respiratory depression if given in doses exceeding an individual’s tolerance; however, the ED is an ideal location to safely monitor patients during methadone initiation.^27^ Previous federal regulations had restricted the use of methadone to treat OUD to licensed OTPs in the outpatient setting.^28^ The Easy Medication Access and Treatment for Opioid Addiction Act improved the flexibility of MOUD by allowing practitioners to dispense up to a three-day supply of narcotics, including methadone, for the purpose of initiating maintenance or detoxification treatment.^29^ ^–^ ^33^

Patients prefer selecting the optimal treatment pathway through shared decision-making, which involves a thorough comparison of the advantages and disadvantages of buprenorphine vs methadone. This approach fosters informed and collaborative healthcare choices and potentially leads to improved outcomes and adherence.^22^ ^,^ ^34^ We found most patients will directly say which treatment pathway they prefer due to past experiences of treatment and precipitated withdrawal. Buprenorphine is advantageous and preferred as it is logistically easier to take: there is less risk of respiratory depression; the patient can receive take-home medications and prescriptions; and care can ultimately be managed by the patient’s primary care physician without daily trips to the methadone clinic for dosing.^19^ However, patients who have experienced buprenorphine-precipitated withdrawal or who cannot tolerate cessation of fentanyl for the required pre-buprenorphine induction time (often 72 hours or longer) may benefit from methadone.^35^

Although surveys from other institutions indicate physicians have more comfort initiating buprenorphine over methadone (88% vs 45%), our experience shows clinicians readily adopting the methadone treatment pathway.^36^ To foster clinician acceptance of ED MOUD, we implemented several strategies: sharing testimonials from our ED peer recovery coaches; facilitating one-on-one discussions between project champions and clinicians; and leveraging direct clinical experience. These approaches align with existing research, which demonstrates increased exposure to impacted populations effectively reduces stigma towards them.^37^ Overcoming these barriers enhanced treatment options through the successful implementation of a methadone initiation pathway for ED patients with OUD.

## LIMITATIONS

Limitations of this study include a small sample size that restricts the generalizability of results and impacts statistical significance. Additionally, the absence of long-term outcome data, such as six-month follow-up metrics, limits insights into the intervention’s effectiveness. The exclusion of patients concurrently enrolled in other treatment programs introduced selection bias, potentially affecting the study’s applicability to the wider OUD population. Finally, individual clinician biases may have influenced both participant selection and treatment choice, potentially affecting study outcomes.

## CONCLUSION

ED initiation of methadone for patients with opioid use disorder is practical, achieves 72-hour follow-up treatment engagement rates comparable to those of buprenorphine treatment, and provides another option for MOUD that benefits some patients.
